# Cerebral Amyloid Angiopathy-Related Inflammation Mimicking Leptomeningeal Metastasis After Resected Lung Adenocarcinoma: A Case Report Highlighting Dynamic Diagnostic Reassessment

**DOI:** 10.3390/curroncol33090562

**Published:** 2026-09-16

**Authors:** Kodai Shigeyama, Yosuke Dotsu, Shunsuke Yoshimura, Takeshi Hiu, Nozomi Ueki, Ryo Futsuki, Satoru Koga, Noritaka Honda, Kazumasa Akagi, Midori Matsuo, Hirokazu Taniguchi, Shinnosuke Takemoto, Hiroshi Mukae

**Affiliations:** 1 Department of Respiratory Medicine, Nagasaki University Hospital, Nagasaki 852-8501, Japan; k.shigeyama@nagasaki-u.ac.jp (K.S.);; 2 Department of Neurology and Strokology, Nagasaki University Hospital, Nagasaki 852-8501, Japan; 3 Department of Neurosurgery, Nagasaki University Graduate School of Biomedical Sciences, Nagasaki 852-8501, Japan; 4 Department of Tumor and Diagnostic Pathology Atomic Bomb Disease Institute, Nagasaki University, Nagasaki 852-8523, Japan; 5 Clinical Oncology Center, Nagasaki University Hospital, Nagasaki 852-8501, Japan; 6 Clinical Research Center, Nagasaki University Hospital, Nagasaki 852-8501, Japan; 7 Clinical Genomics Center, Nagasaki University Hospital, Nagasaki 852-8501, Japan; 8 Nagasaki Harbor Medical Center, Nagasaki 850-8555, Japan

**Keywords:** cerebral amyloid angiopathy-related inflammation, leptomeningeal metastasis, lung adenocarcinoma, brain biopsy, diagnostic reasoning

## Abstract

Cerebral amyloid angiopathy-related inflammation (CAA-ri) is a rare inflammatory subtype of cerebral amyloid angiopathy that may present with leptomeningeal enhancement on magnetic resonance imaging (MRI), thereby mimicking leptomeningeal metastasis (LM) in patients with cancer. We report the case of an 82-year-old woman with a recently resected lung adenocarcinoma who underwent brain MRI following a fall. MRI revealed bilateral asymmetric white matter hyperintensities and multifocal leptomeningeal enhancement, which initially suggested LM. However, preserved neurological function, negative cerebrospinal fluid cytology, and the subsequent identification of numerous cortical and subcortical microbleeds on targeted reassessment with susceptibility-weighted MRI prompted diagnostic reassessment. Brain biopsy demonstrated vascular amyloid deposition with perivascular inflammatory infiltrates, confirming CAA-ri. Because the patient remained neurologically stable, she was managed conservatively, and spontaneous radiological improvement was observed during follow-up. This case highlights the fact that leptomeningeal abnormalities in patients with lung cancer do not invariably represent leptomeningeal metastasis. When clinicoradiological findings are discordant, prompt reassessment using appropriately selected MRI sequences should be integrated with cerebrospinal fluid analysis and multidisciplinary evaluation, with pathological confirmation considered when clinically consequential alternative diagnoses cannot be excluded. This approach may help avoid inappropriate LM-directed therapy.

## Simple Summary

This report describes an older woman who had recently undergone surgery for early-stage lung cancer. A brain MRI performed after a fall showed abnormalities that initially looked like cancer spread to the lining of the brain. However, she had almost no neurological symptoms, no cancer cells were found in the cerebrospinal fluid, and a special MRI sequence later revealed many small brain bleeds. These findings suggested cerebral amyloid angiopathy-related inflammation, a rare inflammatory disease of small brain vessels. Because this condition and leptomeningeal metastasis require very different treatments, a brain biopsy was performed and confirmed the inflammatory disease. The patient remained stable and improved without steroid treatment. This case shows that brain MRI abnormalities in patients with cancer are not always metastases. Careful review of symptoms, cerebrospinal fluid results, appropriate MRI sequences, multidisciplinary discussion, and biopsy when needed can prevent an incorrect diagnosis, unnecessary cancer treatment, and serious treatment delays.

## 1. Introduction

Central nervous system (CNS) abnormalities detected during surveillance or evaluation of neurological symptoms in patients with lung cancer are commonly interpreted as metastatic diseases. Among these, leptomeningeal enhancement on contrast-enhanced magnetic resonance imaging (MRI) frequently raises immediate concerns regarding leptomeningeal metastasis (LM) because of its poor prognosis and important implications for staging, treatment selection, and overall patient management [1]. Although contrast-enhanced MRI is highly sensitive for detecting leptomeningeal disease, its findings are not pathognomonic, and a variety of inflammatory, infectious, and vascular disorders may produce similar radiological appearances [1]. Consequently, an accurate differential diagnosis is essential because misdiagnosis may result in inappropriate staging, unnecessary anticancer therapy, and delayed institution of appropriate treatment. The role of routine preoperative brain MRI in patients with asymptomatic clinical stage I NSCLC, particularly stage IA disease, remains debated because the prevalence of occult brain metastases is low in this population [2]. Nevertheless, preoperative brain imaging may provide a valuable baseline when subsequent neurological or radiological abnormalities arise.

Cerebral amyloid angiopathy-related inflammation (CAA-ri) is a rare immune-mediated inflammatory disorder caused by an autoimmune response against vascular amyloid-β deposition. Typical MRI findings include asymmetric white matter hyperintensities, cortical or subcortical microbleeds, and variable leptomeningeal enhancement [3,4,5]. Although validated clinicoradiological criteria allow the diagnosis of probable CAA-ri without brain biopsy in many appropriately selected patients, histopathological confirmation remains important when diagnostic uncertainty persists or when alternative diagnoses would alter management [6]. Recent systematic reviews have further demonstrated that leptomeningeal enhancement, although less common than white matter abnormalities or cerebral microbleeds, is a recognized manifestation of CAA-ri and may closely mimic LM, particularly in patients with a history of malignancy [3,4,5].

In routine oncology practice, a previous diagnosis of cancer increases the pre-test probability of metastatic disease, and radiological abnormalities are frequently interpreted within this clinical context. Although this approach is often appropriate, it carries the risk of premature diagnostic closure when alternative non-neoplastic disorders present with overlapping imaging features. Rather than relying on a single imaging examination, the accurate diagnosis of CNS lesions often requires the continuous integration of evolving clinical findings, serial neuroimaging, cerebrospinal fluid analysis, and, when necessary, pathological confirmation. Such iterative diagnostic reassessments are particularly important when appropriate management depends on the underlying etiology.

Here, we report a biopsy-proven case of CAA-ri that initially mimicked LM in an 82-year-old woman with resected lung adenocarcinoma. In addition to describing an uncommon neurological disorder, this case illustrates an important diagnostic challenge encountered in thoracic oncology. This demonstrates that not all CNS lesions in patients with lung cancer are metastatic and highlights how serial reassessment integrating clinical presentation, evolving neuroimaging findings, cerebrospinal fluid analysis, multidisciplinary discussion, and histopathological evaluation can redirect diagnostic reasoning, establish the correct diagnosis, and ultimately prevent unnecessary anticancer treatment.

## 2. Case Presentation

**Figure 1:**
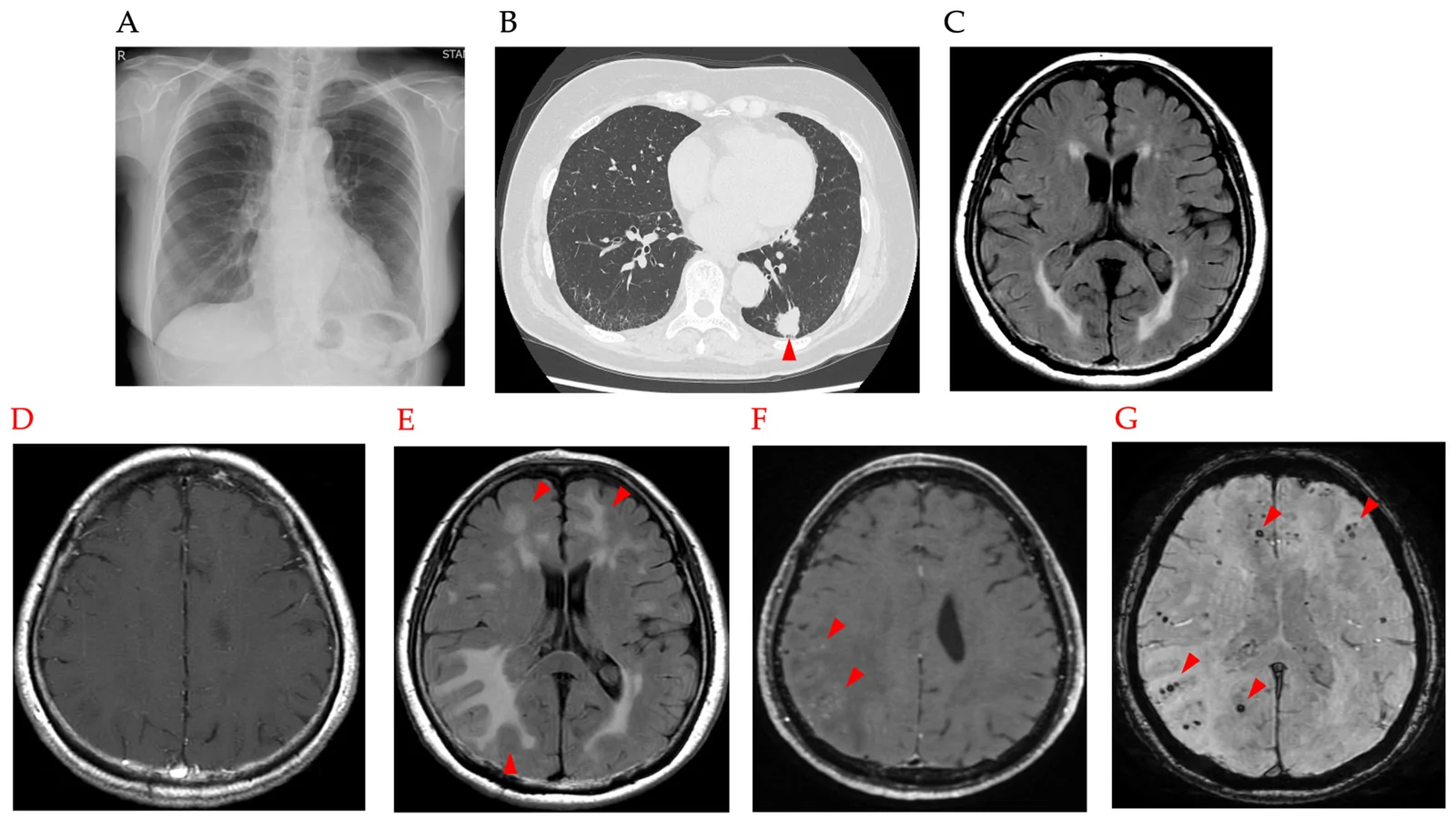
Serial radiological findings illustrating the evolution of the diagnostic assessment. (**A**) Preoperative chest radiograph showing a solitary pulmonary lesion in the left lower lung field. (**B**) Preoperative chest computed tomography demonstrating a left lower lobe lung tumor (arrowhead), which was subsequently resected and pathologically diagnosed as stage IA3 lung adenocarcinoma. (**C**) Preoperative fluid-attenuated inversion recovery (FLAIR) brain magnetic resonance imaging (MRI) demonstrating mild symmetric periventricular white matter hyperintensities considered nonspecific and compatible with age-related small-vessel change; the asymmetric white matter abnormalities observed subsequently were not present. (**D**) Preoperative contrast-enhanced T1-weighted MRI showing no leptomeningeal enhancement or other findings suggestive of leptomeningeal metastasis. (**E**) Post-fall FLAIR MRI demonstrating newly developed bilateral asymmetric cerebral white matter hyperintensities (arrowheads), which were not present on the preoperative FLAIR image. (**F**) Post-fall contrast-enhanced T1-weighted MRI demonstrating newly developed multiple punctate foci of leptomeningeal enhancement (arrowheads), which were absent on the preoperative examination, initially raising concern for leptomeningeal metastasis. (**G**) Follow-up susceptibility-weighted angiography (SWAN) MRI demonstrating numerous cortical and subcortical microbleeds, with representative lesions indicated by arrowheads. The combination of asymmetric white matter hyperintensities and multiple cortical and subcortical microbleeds shifted the leading clinicoradiological diagnosis toward cerebral amyloid angiopathy-related inflammation.

An 82-year-old woman was referred to our hospital after brain magnetic resonance imaging (MRI) revealed unexpected abnormalities. Approximately two weeks earlier, she had experienced an unexplained fall without loss of consciousness or focal neurological symptoms. Because her symptoms were mild, she did not seek immediate medical attention. A mild headache subsequently persisted, prompting brain MRI approximately two weeks after the fall. Three months earlier, she had undergone surgical resection of a papillary-predominant (80%) lung adenocarcinoma with minor acinar (15%) and solid (5%) components, without spread through air spaces (STAS) (pT1cN0M0, pathological stage IA3 according to the ninth edition of the TNM classification), and subsequently started adjuvant tegafur-uracil (UFT) chemotherapy. This treatment was not administered as part of a clinical trial. In Japan, adjuvant UFT is an established postoperative treatment option for patients with completely resected, node-negative lung adenocarcinoma measuring >2 cm [7]. Given the patient’s advanced age, UFT was selected after individualized consideration of the potential benefits and risks of postoperative therapy. Preoperative chest radiography and computed tomography are shown in Figure 1A,B. Preoperative brain fluid-attenuated inversion recovery (FLAIR) MRI demonstrated only mild symmetric periventricular white matter hyperintensities, which were considered nonspecific and compatible with age-related small-vessel changes; the bilateral asymmetric white matter abnormalities observed subsequently were not present (Figure 1C). Preoperative contrast-enhanced T1-weighted MRI showed no leptomeningeal enhancement or other findings suggestive of LM (Figure 1D).

Brain MRI performed after the fall demonstrated newly developed bilateral asymmetric white matter hyperintensities on FLAIR imaging (Figure 1E) and multiple punctate foci of leptomeningeal enhancement on contrast-enhanced T1-weighted imaging (Figure 1F). Apart from mild headache, the patient had no cognitive impairment, seizures, focal neurological symptoms, or constitutional complaints. The neurological examination results were unremarkable.

Given the recent history of lung adenocarcinoma, these imaging findings raised a strong clinical suspicion of LM. The laboratory findings, including serum tumor markers and cerebrospinal fluid (CSF) analysis, are summarized in Table 1. Serum carcinoembryonic antigen (CEA) and soluble interleukin-2 receptor (sIL-2R) levels were within the normal range. Lumbar puncture performed on day 19 after the post-fall brain MRI demonstrated an elevated CSF protein concentration (114 mg/dL) with a normal leukocyte count (2 cells/μL), while CSF cytology showed no malignant cells.

**Figure 2:**
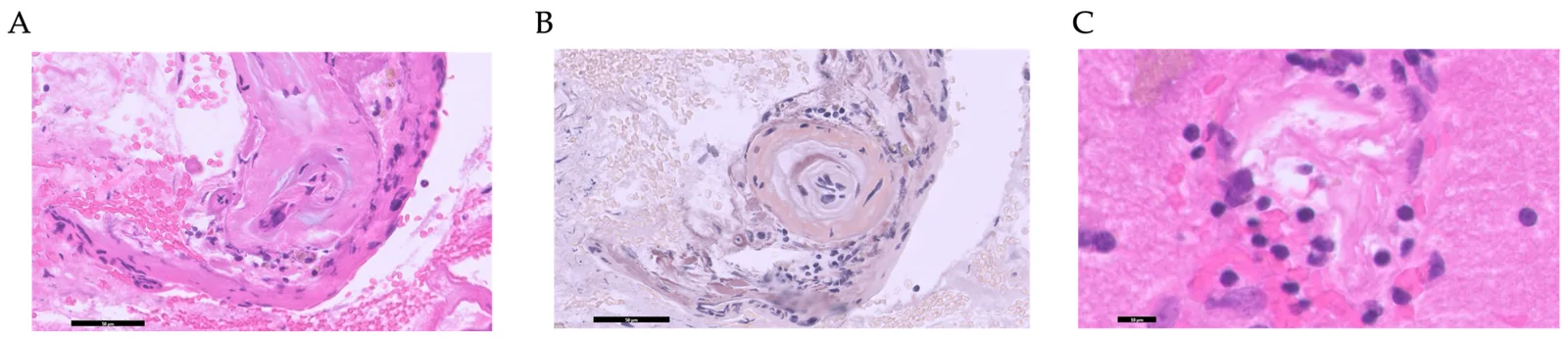
Histopathological findings establishing the diagnosis of cerebral amyloid angiopathy-related inflammation (CAA-ri). (**A**) Hematoxylin and eosin staining showing perivascular inflammatory cell infiltration around small leptomeningeal vessels (original magnification, ×40; scale bar, 20 μm). (**B**) Direct fast scarlet (DFS) staining demonstrating amyloid deposition within the walls of leptomeningeal vessels (original magnification, ×40; scale bar, 20 μm). (**C**) Higher-magnification hematoxylin and eosin image showing focal perivascular inflammatory cell infiltration around a small cortical vessel. Together with the vascular amyloid deposition demonstrated by DFS staining, these findings support the diagnosis of CAA-ri (original magnification, ×100; scale bar, 10 μm).

Although LM remained an important diagnostic consideration, the overall clinicoradiological context, including the early pathological stage of resected lung cancer, preserved neurological function, and negative CSF cytology, did not provide sufficient diagnostic certainty to initiate LM-directed therapy. Therefore, a careful reassessment was performed. Because diagnostic uncertainty persisted, follow-up MRI incorporating susceptibility-weighted angiography (SWAN) was performed 40 days after the post-fall MRI, which demonstrated numerous cortical and subcortical microbleeds that had not been detected in earlier examinations (Figure 1G). The combination of asymmetric white matter hyperintensities and newly recognized cerebral microbleeds increased the likelihood of CAA-ri, fulfilling the clinicoradiological criteria compatible with probable CAA-ri. However, because the patient had recently undergone curative resection for lung adenocarcinoma and a diagnosis of LM would have fundamentally altered the subsequent oncological management, LM could not be confidently excluded despite the clinicoradiological findings. This case was discussed at a multidisciplinary conference involving neurologists, neurosurgeons, and thoracic oncologists. Although repeat CSF cytology was considered, the radiologically abnormal lesion was considered safe for biopsy, and the histopathological differentiation between CAA-ri and LM was expected to directly determine subsequent management. Therefore, tissue diagnosis was prioritized over repeated CSF sampling, and a brain biopsy was performed on day 62 after the post-fall MRI. Histopathological examination revealed amyloid deposition within the vascular walls on direct fast scarlet staining together with prominent perivascular inflammatory cell infiltration, confirming the diagnosis of CAA-ri (Figure 2). Beyond hematoxylin and eosin and direct fast scarlet staining, no additional pathological staining, including amyloid-β immunohistochemistry, was performed because these assays were not available at our institution.

**Figure 3:**
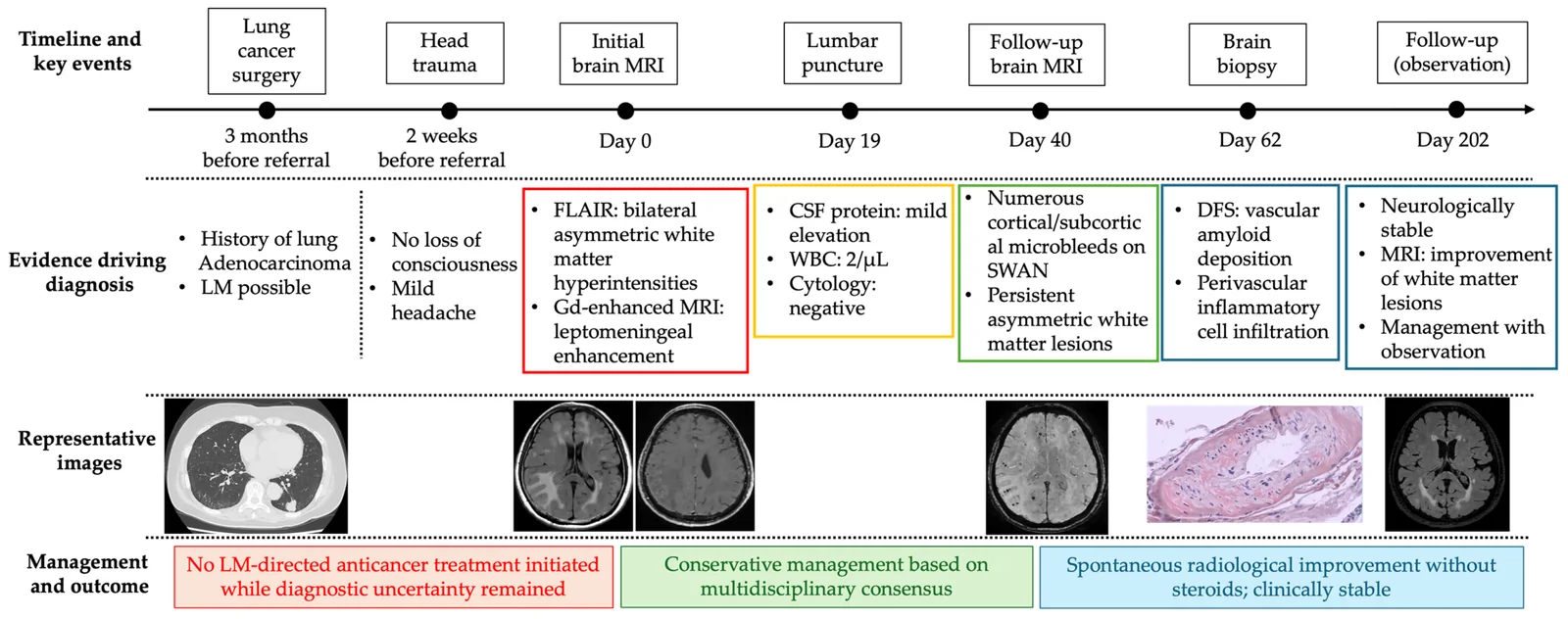
Longitudinal diagnostic assessment and clinical course. The timeline summarizes key clinical events, diagnostic findings, representative images, and management decisions. For the diagnostic timeline shown in this figure, the post-fall brain MRI was defined as day 0; this was not the patient’s first brain MRI, as a preoperative brain MRI had been performed before lung cancer surgery (Figure 1C,D). The unexplained fall occurred approximately two weeks before day 0. Lumbar puncture was performed on day 19, follow-up brain MRI incorporating susceptibility-weighted angiography (SWAN) on day 40, and brain biopsy on day 62. The post-fall brain MRI demonstrated bilateral asymmetric white matter hyperintensities and leptomeningeal enhancement, raising concern for leptomeningeal metastasis. Preserved neurological function and negative cerebrospinal fluid cytology prompted further diagnostic reassessment. Follow-up susceptibility-weighted angiography revealed numerous cortical and subcortical microbleeds with persistent asymmetric white matter lesions, shifting the leading clinicoradiological diagnosis toward cerebral amyloid angiopathy-related inflammation (CAA-ri). Brain biopsy demonstrated vascular amyloid deposition with perivascular inflammatory cell infiltration, establishing the diagnosis of CAA-ri. Leptomeningeal metastasis-directed therapy was deferred pending diagnostic clarification. The patient subsequently remained neurologically stable and showed spontaneous radiological improvement without corticosteroid therapy.

Following the pathological diagnosis, the patient was managed under close clinical observation because she remained neurologically stable without progressive symptoms. Serial follow-up MRI performed 202 days after the post-fall MRI demonstrated spontaneous improvement in white matter hyperintensities without corticosteroid therapy (Figure 3). LM-directed therapy was deferred until the competing diagnoses were resolved, whereas corticosteroid therapy for CAA-ri was not initiated after the diagnosis because of the patient’s minimal symptoms and stable clinical course. Corticosteroid treatment was planned for neurological deterioration and radiological progression.

## 3. Discussion

Leptomeningeal enhancement in patients with lung cancer raises immediate concern for LM, a serious complication with major implications for prognosis, staging, and treatment selection [1,8]. Importantly, in the present case, preoperative brain MRI had been performed before lung cancer resection and showed no leptomeningeal enhancement or other findings suggestive of LM; only mild symmetric periventricular white matter hyperintensities considered compatible with age-related small-vessel change were present (Figure 1C,D). The asymmetric white matter hyperintensities and punctate leptomeningeal enhancement detected on the post-fall MRI were therefore newly developed findings compared with the preoperative examination. However, leptomeningeal enhancement is a radiological pattern rather than a disease-specific finding, and may also occur in infectious, inflammatory, or vascular disorders. Consequently, contrast-enhanced MRI should be interpreted in conjunction with clinical presentation and CSF findings rather than in isolation [8,9]. Likewise, the current EANO-ESMO guidelines recommend that the diagnosis of LM be based on an integrated assessment of neurological manifestations, contrast-enhanced MRI of the neuraxis, and CSF cytology because neither modality alone provides sufficient diagnostic certainty in all patients [8]. Furthermore, a recent systematic review and meta-analysis demonstrated that both MRI and CSF cytology have limited sensitivity despite their relatively high specificity, underscoring the importance of interpreting negative CSF cytology within the overall clinical context and repeated CSF evaluation when appropriate [9]. Thus, although the initial suspicion of LM in our patient was clinically appropriate, the negative CSF cytology reduced but did not eliminate the likelihood of this diagnosis.

Although the initial suspicion of LM was clinically reasonable, several findings were inconsistent with the diagnosis. First, the primary lung adenocarcinoma had been completely resected as pathological stage IA3 without nodal or distant metastases. In addition, the resected tumor was papillary-predominant and lacked STAS, although a minor solid component was present. While these pathological findings cannot exclude subsequent leptomeningeal metastasis, they did not strongly suggest unusually aggressive biological behavior. Although LM cannot be excluded based solely on early pathological stage, it is considerably more common in advanced NSCLC [1]. Second, despite the extensive bilateral white matter abnormalities, the patient remained neurologically intact. Although a normal neurological examination does not exclude LM, the paucity of symptoms relative to the radiological burden adds to the overall clinicoradiological discordance. Third, CSF analysis demonstrated elevated protein levels without pleocytosis and cytology revealed no malignant cells. Finally, the coexistence of multifocal leptomeningeal enhancement with bilateral asymmetric white matter hyperintensities suggests that the imaging findings could not be fully explained by LM alone and raises the possibility of an alternative inflammatory or vascular process. The clinical, radiological, CSF, and histopathological features that aided in the differential diagnosis of LM and CAA-ri are summarized in Table 2. Collectively, these clinicoradiological discrepancies did not exclude LM, but supported further diagnostic clarification before initiating LM-directed therapy.

One of the most instructive aspects of the present case was not simply the repetition of MRI, but also the acquisition and careful review of diagnostically complementary imaging sequences. At initial assessment, asymmetric white matter hyperintensities and multifocal leptomeningeal enhancement in a patient with recently resected lung adenocarcinoma raised concerns regarding LM. However, the early pathological stage of the primary tumor, preserved neurological function, and negative CSF cytology resulted in substantial diagnostic uncertainty. Follow-up susceptibility-weighted imaging revealed numerous cortical and subcortical microbleeds, shifting the leading clinicoradiological diagnosis toward CAA-ri. This case should not be interpreted to support a delayed diagnosis while awaiting the development of cerebral microbleeds. Rather, when clinicoradiological findings remain discordant, prompt reassessment using an optimized MRI protocol, together with CSF evaluation, and when necessary, histopathological confirmation, may facilitate timely diagnostic clarification.

Cerebral microbleeds identified on susceptibility-weighted imaging are diagnostically important because strictly lobar microbleeds and cortical superficial siderosis are key hemorrhagic markers in the clinicoradiological assessment of probable CAA-ri [3,6,10]. These lesions reflect the underlying vascular pathology of CAA-ri, in which amyloid-β deposition is associated with inflammatory activation, blood–brain barrier dysfunction, and vessel-wall injury [11]. Susceptibility-weighted imaging is more sensitive than conventional T2-weighted gradient-recalled echo imaging for the detection of cerebral microbleeds [12,13,14]. In the present case, it remained uncertain whether the microbleeds had newly developed or were already present, but were not sufficiently conspicuous on earlier examination. Regardless, their recognition on susceptibility-weighted imaging provided a more coherent explanation for the combination of asymmetric white matter hyperintensities and leptomeningeal enhancement than LM alone and fundamentally altered the diagnostic assessment.

Another noteworthy aspect of this case was its atypical clinical course. Despite the extensive asymmetric white matter lesions and leptomeningeal enhancement that developed after surgery, the patient remained neurologically intact apart from a mild headache. CAA-ri typically presents with subacute neurological manifestations, including encephalopathy, seizures, focal neurological deficits, and cognitive impairment [3,6]. The relatively indolent clinical course and marked dissociation between the extent of radiological abnormalities and the paucity of neurological symptoms were atypical and contributed to persistent diagnostic uncertainty. These features further support careful longitudinal reassessment before the final diagnosis is established.

Although validated clinicoradiological criteria allow for the diagnosis of probable CAA-ri without brain biopsy in many patients [6], histopathological confirmation remains appropriate when substantial diagnostic uncertainty persists or when clinically important alternative diagnoses cannot be confidently excluded [6,10]. In the present case, follow-up susceptibility-weighted MRI revealed numerous cortical and subcortical microbleeds together with asymmetric white matter hyperintensities, making CAA-ri the leading clinicoradiological diagnosis. Nevertheless, because the patient recently underwent curative resection for lung adenocarcinoma, LM remains a clinically important competing diagnosis that cannot be confidently excluded. This distinction is particularly important because these two conditions require fundamentally different therapeutic strategies and have markedly different prognostic implications. Following multidisciplinary discussions among neurologists, neurosurgeons, neuroradiologists, and thoracic oncologists, a brain biopsy was performed to establish diagnostic certainty. Histopathological examination revealed vascular amyloid deposition with perivascular inflammatory infiltrates, confirming CAA-ri, but excluding pathological evidence of LM. Accordingly, the purpose of brain biopsy in this case was not to establish the diagnosis of CAA-ri itself but to resolve persistent diagnostic uncertainty by excluding a clinically consequential alternative diagnosis that could not be confidently ruled out on clinicoradiological grounds alone. This case illustrates that even in disorders for which validated clinicoradiological criteria are available, brain biopsy may remain justified when the principal clinical question is not confirmation of the leading diagnosis itself but exclusion of an alternative disease with fundamentally different therapeutic implications. CSF amyloid-β biomarkers, including Aβ40 and Aβ42, and amyloid PET were not evaluated in this patient. This represents a limitation of our diagnostic assessment, as these non-invasive approaches might have provided additional evidence supporting cerebral amyloid deposition and potentially reduced the need for an invasive diagnostic procedure. However, CSF Aβ40 and Aβ42 findings in CAA-ri have been inconsistent [15], and neither CSF amyloid-β biomarkers nor amyloid PET would have definitively excluded LM, which remained the clinically consequential alternative diagnosis in this case.

Another notable feature of the present case is the spontaneous radiological improvement observed without corticosteroid therapy. Immunosuppressive treatment, usually with corticosteroids, is generally considered for symptomatic or progressive CAA-ri; however, spontaneous clinical and radiological improvements have occasionally been reported in patients with mild or minimally symptomatic disease [5]. In the present case, conservative management was selected because the patient remained neurologically stable and showed no evidence of radiological deterioration after pathological diagnosis. This favorable course should not be interpreted as supporting routine observation for CAA-ri; rather, it highlights the need to individualize management according to neurological severity, radiological evolution, and overall clinical context. When an initial conservative approach is selected, close clinical and radiological surveillance is essential to allow the prompt initiation of treatment if deterioration occurs.

Beyond illustrating the uncommon presentation of CAA-ri, this case highlights the broader principle of clinical diagnostic reasoning. Diagnostic errors often arise not from insufficient medical knowledge, but from cognitive biases that influence clinical judgment [16,17]. Among these, premature diagnostic closure, the tendency to accept an initial diagnosis before adequately considering conflicting evidence, is a well-recognized contributor to diagnostic error [16]. In oncological practice, where a history of malignancy appropriately increases the pre-test probability of metastatic disease, this cognitive bias may be particularly difficult to avoid. Our case demonstrates that diagnostic assessment should be continuously refined through the integration of clinical presentation, laboratory findings, appropriately selected neuroimaging sequences, and, when necessary, histopathological evidence, rather than relying on the initial radiological impression alone. Maintaining diagnostic flexibility is particularly important when competing diagnoses have different therapeutic implications.

## 4. Conclusions

Not all central nervous system lesions in patients with lung cancer are metastatic. When clinicoradiological findings are discordant, diagnostic assessments should be continuously refined through the integration of appropriately selected neuroimaging sequences, cerebrospinal fluid evaluation, multidisciplinary discussions, and, when necessary, histopathological confirmation. Susceptibility-sensitive MRI may reveal diagnostically decisive hemorrhagic markers when considering CAA-ri. This iterative yet timely diagnostic approach may prevent premature diagnostic closure, avoid inappropriate LM-directed therapy, and improve patient-centered care.

## Figures and Tables

**Table 1:** Laboratory and cerebrospinal fluid findings at initial evaluation.

Parameter	Reference Range	Result	Diagnostic Implication
Blood tests			
WBC count (/μL)	3300–8600	5100	No evidence of systemic inflammation
CRP (mg/dL)	0.00–0.14	0.04	No evidence of active inflammation
CEA (ng/mL)	≤5.0	1.3	No biochemical evidence supporting recurrence
sIL-2R (U/mL)	121–613	300	Less supportive of lymphoma
Cerebrospinal fluid			
Opening pressure (mmH_2_O)	50–180	185	Borderline elevation without marked intracranial hypertension
WBC count (/μL)	≤5	2	No pleocytosis
Protein (mg/dL)	≤45	114	Mild elevation; nonspecific finding
Glucose (mg/dL)	45–80	60	Preserved CSF glucose level
Cytology	-	Negative	No malignant cells identified

Abbreviations: WBC, white blood cell; CRP, C-reactive protein; CEA, carcinoembryonic antigen; sIL-2R, soluble interleukin-2 receptor.

**Table 2:** Clinical and radiological features differentiating Cerebral Amyloid Angiopathy-Related Inflammation (CAA-ri) from Leptomeningeal Metastasis.

Feature	LM	CAA-ri	Present Case	Diagnostic Implication
Typical clinical presentation	Progressive neurological deficits, headache, cranial neuropathies, seizures	Acute or subacute encephalopathy, headache, seizures, focal deficits	Mild headache only; preserved neurological function	Mild symptoms were less consistent with LM and prompted diagnostic reassessment
White matter hyperintensities	Usually absent or nonspecific	Asymmetric vasogenic white matter hyperintensities	Bilateral asymmetric white matter hyperintensities	Supported the possibility of CAA-ri but were not diagnostic
Leptomeningeal enhancement	Typical MRI finding	May be present	Present	Initially favored LM
Cortical/subcortical microbleeds	Uncommon	Characteristic imaging feature	Numerous lobar cortical and subcortical microbleeds	Strongly shifted the differential diagnosis toward CAA-ri
CSF cytology	Frequently positive	Negative for malignant cells	Negative	Reduced the likelihood of LM but did not exclude it
CSF protein	Often elevated	May be elevated	Elevated	Nonspecific finding
Histopathology	Malignant leptomeningeal infiltration	Vascular amyloid deposition with perivascular inflammation	Vascular amyloid deposition with perivascular inflammatory infiltrates	Established the definitive diagnosis of CAA-ri

Abbreviations: CAA-ri, cerebral amyloid angiopathy-related inflammation; LM, leptomeningeal metastasis; CSF, cerebrospinal fluid; MRI, magnetic resonance imaging.

## Data Availability

The original contributions presented in this study are included in the article. Further inquiries can be directed to the corresponding author.
